# Deep Anterior Lamellar Keratoplasty Versus Penetrating Keratoplasty in Keratoconus: A Retrospective Study Using Coarsened Exact Matching

**DOI:** 10.18502/jovr.v20.15222

**Published:** 2025-09-09

**Authors:** Farid Karimian, Kiana Hassanpour, Farbod Semnani, Sepehr Feizi, Mohammad Ali Javadi, Amir Faramarzi, Mohammadreza Jafarinasab

**Affiliations:** ^1^Ophthalmic Research Center, Research Institute for Ophthalmology and Vision Science, Shahid Beheshti University of Medical Sciences, Tehran, Iran; ^2^Department of Ophthalmology, Labbafinejad Medical Center, Shahid Beheshti University of Medical Sciences, Tehran, Iran; ^3^National Center for Health Insurance Research, Tehran, Iran; ^4^School of Medicine, Tehran University of Medical Sciences, Tehran, Iran

**Keywords:** Coarsened Exact Matching, Deep Anterior Lamellar Keratoplasty, Graft Failure, Graft Rejection; Keratoconus, Penetrating Keratoplasty

## Abstract

**Purpose:**

To compare the graft survival rates and visual outcomes in patients with keratoconus (KCN) undergoing deep anterior lamellar keratoplasty (DALK) versus penetrating keratoplasty (PKP) and identify possible risk factors.

**Methods:**

This retrospective longitudinal study enrolled patients with KCN who underwent corneal transplantation at Labbafinejad Medical Center (Tehran, Iran) between 2006 and 2016. We utilized a one-to-one coarsened exact matching (CEM) approach. Matching considered the recipient's sex, donor age, and the presence and extent of corneal neovascularization in the recipient.

**Results:**

A total of 213 patients with KCN who underwent PKP (49.8%; *n* = 106) or DALK (50.2%; *n* = 107) were enrolled. After CEM was performed, 67 patients in the PKP group were well-matched with 67 patients in the DALK group. There was no significant difference in terms of baseline characteristics between the two groups. Kaplan-Meier survival analysis showed that the percentage of patients free of graft rejection in a one- and three-year follow-up was 78.7% and 74.5% in the PKP group, and 95.3% and 95.3% in the DALK group, respectively. On univariate analysis, the strongest risk factor predictive of graft rejection was corneal vascularization in both the PKP and DALK groups, followed by recipient age only in the PKP group and dry eye disease only in the DALK group. In a multivariate Cox regression analysis, PKP was identified as an independent predictor of graft rejection, but not of graft failure. The DALK and PKP groups were comparable in terms of postoperative best-corrected visual acuity (BCVA; *P = *0.48) and suture complications (*P = *0.87).

**Conclusion:**

In patients with KCN, DALK demonstrated comparable outcomes to PKP in terms of BCVA, but showed superiority in graft rejection-free survival. Corneal vascularization was the major recipient risk factor for graft rejection and failure.

##  INTRODUCTION

Over the past half century, penetrating keratoplasty (PKP) has been the preferred surgical procedure for advanced keratoconus (KCN).^[1,2]^ According to a study in Iran, KCN is the most common indication of PKP.^[3]^ The more recent method for treating KCN is deep anterior lamellar keratoplasty (DALK), in which the endothelium and the Descemet membrane of the host are preserved, and the rest of the corneal stroma is transplanted.^[4,5]^

Transplant rejection, although treatable, is considered to be the most important cause of transplant failure. Several factors have been reported in connection with transplant rejection. Most studies have suggested that host bed vascularity is a significant risk factor. Other risk factors include the age of the host, ABO blood group mismatch, history of previous transplantation, anterior iris synechiae, the number of vascularized host quadrants, large size of the donor graft, suture-related complications like vascularization and loose sutures, corneal epithelial defects, history of glaucoma, history of herpes simplex, and donor related factors.^[6,7,8,9,10]^

In recent years, DALK has evolved to become a serious contender for the PKP method. Although this surgical technique has been in use for many years, it was not widely adopted due to poor visual outcomes. However, surgeons increasingly favor this method owing to the introduction of the big-bubble technique by Anwar et al and advances in surgical instruments, which improve visual and refractive outcomes.^[11,12,13,14]^ The choice between DALK and PKP as surgical modalities for patients with KCN depends on several factors, including the severity of the disease, corneal thickness, and surgeon expertise. Both procedures have advantages and limitations. In general, DALK is preferred in cases where the disease primarily affects the front layers of the cornea, such as cases with mild to moderate KCN. DALK offers the advantage of reduced risk of graft rejection and better long-term graft survival compared to PKP. It also eliminates the risk of endothelial rejection and associated complications.^[15]^ Furthermore, PKP may be preferred when there is significant corneal scarring or stromal thinning, or when the endothelial layer is compromised. However, PKP carries a higher risk of graft rejection and endothelial cell loss compared to DALK.^[16]^

Several studies have compared DALK and PKP in terms of their clinical outcomes and transplant rejection rates, yielding varying results.^[17,18,19,20,21,22]^ Considering the high rate of corneal transplantation in referral centers, a procedure which is expensive and time-consuming for both patients and these centers, it is important to investigate the factors contributing to corneal transplant rejection. Therefore, the present study compared the incidence of transplantation failure and the risk factors affecting transplant rejection and failure among patients with KCN who had undergone either DALK or PKP at Labbafinejad Tertiary Referral Center in Tehran.

##  METHODS 

This retrospective longitudinal study enrolled patients with KCN who underwent corneal transplantation at Labbafinejad Medical Center between 2006 and 2016. The study protocol was approved by the institutional review board of the Ophthalmic Research Center, affiliated with Shahid Beheshti University of Medical Sciences, and adhered to the tenets of the Declaration of Helsinki.

The inclusion criteria consisted of KCN patients who underwent PKP or DALK for the first time. Patients with a repeat graft and a follow-up of less than six months were excluded from the study.

To enhance the rigor of our study, we utilized a one-to-one coarsened exact matching (CEM) approach, a robust methodology in causal inference literature. Matching considered the recipient's sex, donor age (using an automatic binning algorithm based on Sturge's rule), and the presence and extent of recipient corneal neovascularization.^[23]^

We recorded the initial presentation of clinical data, demographics, past medical history, surgical complications, and follow-up data regarding graft rejection and failure. The data-gathering tool in this study was a researcher-designed form which included the following items: patient's sex and age at the time of transplantation, the recipient and donor corneal diameters, fresh versus preserved donor cornea, history of severe ocular allergy, recipient's corneal vascularization by quadrant, repeat transplant, occurrence and timing of graft rejection, and the time interval until complete suture removal. The data also included best-corrected visual acuity (BCVA) and refractive errors at the last examination, complications (epithelial problems, suture-associated problems, cataracts, and increased intraocular pressure), secondary surgical interventions, transplant transparency, and dry eye. Other variables included the presence of comorbidities such as glaucoma or diabetes, the duration of steroid drop administration, the causes of transplant rejection, the type of corneal transplant rejection (epithelial, stromal, or endothelial), and the patient's immune system status.

The diagnosis of KCN was based on the clinical and slit lamp findings (corneal ectasia, stromal thinning, Fleischer ring, and Vogt's striae). The diagnosis was confirmed using topographic findings (TMS-4 Topography Modeling System, Tomey, Nagoya, Japan) or corneal tomographic findings (Orbscan II, Bausch & Lomb, Rochester, NY, USA). After the diagnosis of KCN was established, the patient was considered a candidate for corneal transplantation only if the patient did not achieve acceptable vision after using glasses, was not able to use a rigid gas permeable (RGP) or mini-scleral (MSD) contact lens for any reason, and was not a suitable candidate for the insertion of intracorneal rings.

Eligible patients underwent either DALK or PKP surgery by one of the corneal surgeons. Postoperative examinations were performed one and three days after surgery, followed by periodic examinations until complete epithelial closure was achieved. Subsequent examinations were conducted one month later, then every two months for up to 18 to 24 months. After surgery, 1% betamethasone eye drops (Sina Daru, Tehran, Iran) were prescribed four times daily and tapered within three months. Removal of stitches began four months postoperatively if the amount of achieved astigmatism exceeded 4 diopters, and all sutures were eventually removed, typically within two years postoperatively. At the time of follow-up, complications and graft rejection were confirmed by two corneal specialists.

In the present study, transplant rejection in patients undergoing DALK was classified based on the layer involved in the immune reaction: epithelial, subepithelial, and stromal. In patients undergoing PKP, we considered endothelial immune reactions in addition to the aforementioned types of rejections. Upon occurrence of these reactions, treatment included frequent steroid drops with or without oral steroids.

Transplant failure was considered in corneas with dense haziness (opacity), high final astigmatism, severe edema, vascularization, trauma, and infection, which caused low visual acuity and irreversible changes within three to six months. Graft opacity was graded from 0 to 4 as follows: absolutely transparent (grade 0), minimally opaque but easily visible iris vessels (grade 1), moderately opaque with iris vessels still visible (grade 2), opaque with iris vessels totally invisible (only pupil margin visible; grade 3), and completely opaque (grade 4).^[24]^

Corneal interface vascularization—a serious sight-threatening complication of corneal allografts and characterized by the presence of neovascularization in the donor–recipient interface^[25]^—was recorded as either superficial or deep vascularization, categorized by the number of involved quadrants.

We determined the presence and severity of post-transplantation dry eye based on the reduction in tear lake height and the extent of punctate epithelial erosions: absent (normal cornea), mild ( < 1/3 of corneal surface), moderate (1/3–1/2 of corneal surface), and severe ( > 1/2 of corneal surface). The presence of filamentary keratitis on the corneal surface, which required occlusion of the lacrimal punctum, and the need for tarsorrhaphy during follow-up were categorized as severe.

The main outcomes were defined as one- and three-year graft rejection and survival rates. The secondary outcomes were postoperative BCVA and the occurrence of complications.

### Statistical Analysis 

CEM was used to control for imbalance and confounding bias by approximately exact-matching the patients on previously established risk factors for graft failure (primary outcomes) and covariates associated with treatment assignment.^[23,26,27]^

Data analyses were done using Stata version 16 (StataCorp, College Station, TX, USA). All analyses were performed on cases with available data for the variables of interest (excluding missing data). The total number of subjects for any given analysis may therefore be less than the total cohort size. The results of the Shapiro-Wilk test for normally distributed data, along with the histogram and Q-Q plots, indicated that the continuous variables did not follow a normal distribution. Thus, comparisons of patient demographics and pre- and postoperative details between the DALK and PKP groups were performed using the Mann–Whitney U test for continuous variables, the Pearson's Chi-square test (or the Chi-square exact test) for categorical variables, and Cox regression for time-to-event variables. The chi-square test for trend was used to assess the association between the severity of postoperative dry eye and graft rejection. The Wilcoxon signed-rank test was used to compare baseline and postoperative visual and refractive parameters between the two study groups. Kaplan-Meier survival graphs and a log-rank test were used to compare rejection-free graft survival and graft survival rates between the study groups. A *P *value of *<* 0.05 was considered statistically significant. All reported *P *values are two-sided.

##  RESULTS

### Comparison of PKP and DALK in the Unmatched Cohort

The baseline characteristics, perioperative outcomes, and operative details of the patients undergoing PKP and DALK are summarized in Tables 1–3. A total of 213 patients with KCN undergoing primary keratoplasty were enrolled in this study. Of this population, 50.2% (*n* = 107) underwent DALK and 49.8% (*n* = 106) underwent PKP. According to the results, PKP was associated with a significantly higher proportion of female patients, younger donors, and a higher rate of neovascularization, both in a single quadrant of the cornea and in multiple quadrants. In the unmatched cohort, the follow-up period ranged from 6 to 96 months, with a mean of 38.5 ± 24.6 months in the PKP group and 38.1 ± 21.9 months in the DALK group (*P* = 0.55). The big bubble technique was implemented in all DALK surgeries.

A history of vernal keratoconjunctivitis (VKC) was present in 17 eyes in the PKP group (16.0%) and 14 eyes in the DALK group (13.1%; *P *= 0.54). The quality of donor grafts was significantly different between the two groups, as grafts with a higher rate of mild to severe opacity were transplanted to the eyes in the DALK group. Other preoperative characteristics, including donor trephination size, duration of topical steroid administration, suturing technique, and time to suture removal, were comparable between the two study groups.

**Table 1:** Comparison of baseline characteristics between patients undergoing DALK versus PKP

		**Before CEM**			**After CEM**			**Test**
		**DALK (** ***N*** ** = 107)**	**PKP (** ***N*** ** = 106)**	***P*** **-value**	**DALK (** ***N*** ** = 67)**	**PKP (** ***N*** ** = 67)**	***P*** **-value**	
Recipient age (yrs)		25.0 (21.0 to 30.0)	28.0 (21.0 to 35.0)	0.13	26.7 (21 to 29)	28.2 (20 to 33)	0.30	Wilcoxon rank-sum
Recipient sex, *n* (%)	Female	53 (49.5%)	73 (68.9%)	< 0.01	40 (59.7)	40 (59.7)	1.00	Chi-square
	Male	54 (50.5%)	33 (31.1%)		27 (40.3)	27 (40.3)		
Past medical and surgical history, *n* (%)	No	98 (91.6%)	102 (96.2%)	0.09	60 (89.5%)	64 (95.5%)	0.21	Chi-square exact
	Cataract surgery	1 (0.9%)	0 (0.0%)		1 (1.5%)	0 (0.0%)		
	CXL	0 (0.0%)	1 (0.9%)		0 (0.0%)	0 (0.0%)		
	ICRS Surgery	4 (3.7%)	0 (0.0%)		3 (4.5%)	0 (0.0%)		
	RGP lenses	4 (3.7%)	2 (1.9%)		3 (4.5%)	2 (3%)		
	Strabismus	0 (0.0%)	1 (0.9%)		0 (0.0%)	1 (1.5%)		
								
Donor age (yr)		45.0 (30.0 to 53.0)	38.0 (28.0 to 50.0)	0.03	39.7 (30.0 to 50.0)	39.2 (30.0 to 50.0)	0.75	Wilcoxon rank-sum
Donor sex, *n* (%)	Female	83 (77.6%)	84 (83.2%)	0.31	54 (80.6%)	56 (83.6%)	0.65	Chi-square
	Male	24 (22.4%)	17 (16.8%)		13 (19.4%)	11 (16.4%)		
History of trauma, *n* (%)		2 (1.9%)	0 (0.0%)	0.16	1 (1.5%)	0 (0.0%)	1.00	Chi-square exact
Data are presented as median (IQR) for continuous measures and as number (%) for categorical measures. CEM, coarsened exact matching; CXL, corneal cross-linking; DALK, deep anterior lamellar keratoplasty; ICRS, intrastromal corneal ring segments; PKP, penetrating keratoplasty; RGP, rigid gas permeable yrs, years								

**Table 2:** Comparison of pre-, intra-, and postoperative parameters between keratoconus patients who underwent DALK versus PKP

		**Before CEM**			**After CEM**			**Test**
		**DALK (** ***N*** ** = 107)**	**PKP (** ***N*** ** = 106)**	***P*** **-value**	**DALK (** ***N*** ** = 67)**	**PKP (** ***N*** ** = 67)**	***P*** **-value**	
Donor trephination size (IQR), mm		8.3 (8.0 to 8.3)	8.3 (8.0 to 8.3)	0.17	8.3 (8.0 to 8.3)	8.3 (8.0 to 8.3)	0.1	Wilcoxon rank-sum
Surgery duration, *n* (%) minutes	Optimum ( < 85)	42 (42.0%)	56 (54.9%)	0.07	28 (44.4%)	34 (53.1%)	0.33	Chi-square
	High ( ≥ 85)	58 (58.0%)	46 (45.1%)		35 (55.6%)	30 (46.9%)		
Donor graft opacity	Clear	75 (70.1%)	90 (84.9%)	0.03	50 (74.6%)	56 (83.6%)	0.35	Chi-square exact
	Mild	17 (15.9%)	12 (11.3%)		9 (13.4%)	9 (13.4%)		
	Moderate	10 (9.3%)	4 (3.8%)		6 (9.0%)	2 (3.0%)		
	Severe	4 (3.7%)	0 (0.0%)		1 (1.5%)	0 (0.0%)		
	Keloid	1 (0.9%)	0 (0.0%)		1 (1.5%)	0 (0.0%)		
Suture type	Interrupted	59 (56.7%)	41 (39.8%)	0.01	38 (56.7%)	28 (43.8%)	0.13	Chi-square exact
	Combined	45 (43.3%)	59 (57.3%)		29 (43.3%)	34 (53.1%)		
	Single running	0 (0.0%)	3 (2.9%)		0 (0.0%)	2 (3.1%)		
Duration of steroid therapy, *n* (%) months	≤ 4	1 (1.1%)	1 (1.1%)	0.24	1 (1.8%)	1 (1.8%)	0.17	Chi-square exact
	5–8	17 (18.9%)	24 (25.5%)		10 (17.9%)	15 (26.8%)		
	9–12	21 (23.3%)	29 (30.9%)		12 (21.4%)	18 (32.1%)		
	≥ 12	51 (56.7%)	40 (42.6%)		33 (58.9%)	22 (39.2%)		
Time to suture removal, months		19.0 (6.6)	18.4 (6.7)	0.48	18.7 (6.4)	18.8 (6.5)	0.89	Wilcoxon rank-sum
Mean follow-up time (SD)		38.1 (21.9)	38.5 (24.6)	0.55	35.7 (20.1)	39.4 (24.5)	0.77	Wilcoxon rank-sum
Surgeon	Attending	68 (63.6%)	63 (59.4%)	0.54	42 (62.7%)	39 (58.2%)	0.60	Chi-square
	Fellow of cornea	39 (36.4%)	43 (40.6%)		25 (37.3%)	28(41.8%)		
Neovascularization area, *n* (%)	Absent	10 (9.3%)	79 (74.5%)	< 0.01	61 (91%)	61 (91%)	1.00	Chi-square exact
	One quadrant	4 (3.7%)	22 (20.8%)		6 (9%)	6 (9%)		
	Two to fourquadrants	1 (0.9%)	5 (4.7%)		0 (0%)	0 (0%)		
Descemet membrane	Intact	91 (94.4%)	100 (94.3%)	0.03	64 (95.5%)	67 (100.0%)	0.24	Chi-square exact
	Detachment	6 (5.6%)	0 (0.0%)		3 (4.5%)	0 (0.0%)		
Data are presented as median (IQR) for continuous measures and as number (%) for categorical measures, except for follow-up time, which is reported as mean (SD). CEM, coarsened exact matching; DALK, deep anterior lamellar keratoplasty; PKP, penetrating keratoplasty; SD, standard deviation								

**Table 3:** Type of graft rejection between the two study groups

		**Before CEM**			**After CEM**			**Test**
		**DALK (** ***N*** ** = 107)**	**PKP (** ***N*** ** = 106)**	***P*** **-value**	**DALK (** ***N*** ** = 67)**	**PKP (** ***N*** ** = 67)**	***P*** **-value**	
Rejection type	Epithelial	1 (0.9%)	2 (1.9%)	< 0.001	0 (0.0%)	1 (1.5%)	< 0.01	Chi-square exact
	Subepithelial	2 (1.9%)	10 (9.4%)		0 (0.0%)	5 (7.5%)		
	Stromal	3 (2.8%)	2 (1.9%)		2 (3.0%)	1 (1.5%)		
	Endothelial	0 (0.0%)	19 (17.9%)		0 (0.0%)	7 (10.5%)		
Data are presented as median (IQR) for continuous measures and as number (%) for categorical measures. CEM, coarsened exact matching; DALK, deep anterior lamellar keratoplasty; PKP, penetrating keratoplasty.								

**Table 4:** Comparison of the preoperative and postoperative best-corrected visual acuity (BCVA) in the PKP and DALK groups

	**Entire unmatched cohort**			**Coarsened exact matching**			**Test**
	**DALK (** ***N*** ** = 107)**	**PKP (** ***N*** ** = 106)**	***P*** **-value**	**DALK (** ***N*** ** = 67)**	**PKP (** ***N*** ** = 67)**	***P*** **-value**	
Preoperative best corrected visual acuity, LogMAR	1.0 (0.7 to 2.1)	2.0 (1.0 to 2.3)	0.01	1.0 (0.83 to 2.1)	2.0 (1.0 to 2.3)	0.09	Mann–Whitney U test
Postoperative best-corrected visual acuity, LogMAR	0.22 (0.1 to 0.4)	0.22 (0.1 to 0.4)	0.75	0.22 (0.1 to 0.3)	0.22 (0.1 to 0.4)	0.48	Mann–Whitney U test
Data are presented as median (IQR) for continuous measures and as number (%) for categorical measures. CEM, coarsened exact matching; DALK, deep anterior lamellar keratoplasty; PKP, penetrating keratoplasty; LogMAR, logarithm minimum angle of resolution							

**Table 5:** Complications in the DALK versus PKP groups during the study period.

		**Before CEM**			**After CEM**			**Test**
		**DALK (** ***N*** ** = 107)**	**PKP (** ***N*** ** = 106)**	***P*** **-value**	**DALK (** ***N*** ** = 67)**	**PKP (** ***N*** ** = 67)**	***P*** **-value**	
Suture complications	No complication	81 (75.7%)	79 (74.5%)	0.97	51 (76.1%)	48 (71.6%)	0.87	Chi-square exact
	Loose	10 (9.3%)	12 (11.3%)		6 (9.0%)	9 (13.4%)		
	Eroded	7 (6.5%)	6 (5.7%)		5 (7.4%)	5 (7.5%)		
	Embedded	1 (0.9%)	1 (0.9%)		1 (1.5%)	0 (0.0%)		
	Re-suture	4 (3.7%)	6 (5.7%)		2 (3.0%)	3 (4.5%)		
	Tight	2 (1.9%)	1 (0.9%)		0 (0.0%)	1 (1.5%)		
	Vascularization	2 (1.9%)	1 (0.9%)		2 (3.0%)	1 (1.5%)		
Transplant infiltration	No	94 (87.9%)	90 (84.9%)	0.70	60 (89.5%)	59 (88.0%)	1.00	Chi-square exact
	Keratitis	7 (6.5%)	8 (7.6%)		2 (3.0%)	3 (4.5%)		
	Ulcer	2 (1.9%)	1 (0.9%)		2 (3.0%)	1 (1.5%)		
	Stich abscess	4 (3.7%)	7 (6.6%)		3 (4.5%)	4 (6.0%)		
Post-surgical glaucoma	No	106 (99.1%)	103 (97.2%)	0.18	66 (98.5%)	66 (98.5%)	1.00	Chi-square exact
	Glaucoma	0 (0.0%)	3 (2.8%)		0 (0.0%)	1 (1.5%)		
	Suspect for glaucoma	1 (0.9%)	0 (0.0%)		1 (1.5%)	0 (0.0%)		
Post-surgical uveitis		2 (1.9%)	1 (0.9)	0.50	1 (1.5%)	1 (1.5 %)	1.00	Chi-square exact
Persistent epithelial defect		1 (0.9%)	1 (0.9%)	1.00	0 (0.0%)	0 (0.0%)	1.00	Chi-square exact
Dry eye disease, *n* (%)	Absent	75 (70.1%)	77 (72.6%)	0.60	47 (70.2%)	48 (71.6%)	0.69	Chi-square exact
	Mild	11 (10.3%)	14 (13.2%)		7 (10.4%)	10 (14.9%)		
	Moderate	8 (7.5%)	4 (3.8%)		6 (9.0%)	3 (4.5%)		
	Severe	13 (12.1%)	11 (10.4%)		7 (10.4%)	6 (9.0%)		
Data are presented as median (IQR) for continuous measures and as number (%) for categorical measures. CEM, coarsened exact matching; DALK, deep anterior lamellar keratoplasty; PKP, penetrating keratoplasty.								

**Table 6:** Comparison between time to first graft rejection and time to graft failure for keratoconus eyes that underwent PKP versus DALK

		**Before CEM**			**After CEM**			
		**DALK**	**PKP**	***P*** **-value**	**DALK**	**PKP**	***P*** **-value**	**Test**
Duration to the first rejection episode, months		18.5 (5 to 40)	11.5 (6.5 to 22)	0.68	22.5 (4 to 46)	8.5 (6 to 12)	0.65	Wilcoxon rank-sum
More than one rejection episodes, *n* (%)		0 (0.0%)	7 (6.7%)	< 0.001	0 (0.0%)	3 (5.3%)	< 0.001	Chi-square exact
Time to first graft rejection (%) (95% CI)	1-year survival rate	93.2 (86.3 to 96.7)(95 eyes)*	79.0 (69.6 to 85.8)(82 eyes)	< 0.001	95.3 (89.6 to 99.8)(61 eyes)	78.7 (69.8 to 89.6)(50 eyes)	0.02	Log-rank
	3-year survival rate	91.7 (83.9 to 95.8)(40 eyes)	64.3 (52.9 to 73.6)(31 eyes)		95.3 (86.2 to 98.5)(25 eyes)	74.5 (60.1 to 83.9)(22 eyes)		
	5-year survival rate	76.4 (60.0 to 86.8)(16 eyes)	55.5 (41.7 to 67.2)(17 eyes)		84.3 (60.9 to 94.3)(14 eyes)	69.9 (53.7 to 81.3)(14 eyes)		
Graft survival rate (%) (95% CI)	1-year survival rate	97.1 (91.2 to 99.1)(96 eyes)	97.0 (91.0 to 99.0)(87 eyes)	0.71	98.5 (89.6 to 99.8)(61 eyes)	96.7 (87.5 to 99.2)(53 eyes)	0.71	
	3-year survival rate	95.5 (88.1 to 98.3)(40 eyes)	94.6 (87.4 to 97.7)(33 eyes)		98.5 (89.6 to 99.8)(25 eyes)	94.8 (84.8 to 98.3)(22 eyes)		
	5-year survival rate	85.9 (69.9 to 93.7)(16 eyes)	94.6 (87.4 to 97.7)(18 eyes)		87.1 (60.9 to 96.2)(10 eyes)	94.8 (84.8 to 98.3)(14 eyes)		
${}^{*}$ Number of eyes at risk. Data are presented as median (IQR) for continuous measures and as number (%) for categorical measures. CEM, coarsened exact matching; DALK, deep anterior lamellar keratoplasty; PKP, penetrating keratoplasty.								

**Table 7:** Proportion of postoperative dry eye disease between the two study groups and its association with graft rejection

**Before CEM**									
		**DALK (** ***N*** ** = 107)**			**PKP (** ***N*** ** = 106)**				**Test**
		Graft rejection		*P*-value	Graft rejection		*P*-value		Chi-square test for trend
		**No**	**Yes**	< 0.01	**No**	**Yes**	<@0.95		
Dry eye disease, *n* (%)	Absent	69 (74.2%)	6 (42.9%)		52 (76.5%)	25 (65.8%)			
	Mild	9 (9.7%)	2 (14.3%)		6 (8.8%)	8 (21.0%)	<@		
	Moderate	7 (7.5%)	1 (7.1%)		1 (1.5%)	3 (7.9%)	<@		
	Severe	8 (8.6%)	5 (35.7%)		9 (13.2%)	2 (5.3%)			
**After CEM**									
		**DALK (** ***N*** ** = 67)**			**PKP (** ***N*** ** = 67)**				
	Absent	46 (75.4%)	1 (16.6%)	< 0.001	37 (75.5%)	11 (61.1%)	<@0.97		
	Mild	6 (9.8%)	1 (16.6%)		5 (10.2%)	5 (27.8%)	<@		
	Moderate	5 (8.2%)	1 (16.6%)		1 (2.0%)	2 (11.1%)			
	Severe	4 (6.6%)	3 (50.2%)		6 (12.3%)	0 (0.0%)			
Data are presented as median (IQR) for continuous measures and number (%) for categorical measures. CEM, coarsened exact matching; DALK, deep anterior lamellar keratoplasty; PKP, penetrating keratoplasty. yrs, years									

### Comparison of the PKP and DALK in the Matched Cohort 

After 1:1 CEM, 67 patients in the PKP group were well-matched with 67 patients in the DALK group. There was no significant difference in the baseline characteristics between the two groups. The follow-up periods were 39.4 ± 24.5 months and 35.7 ± 20.1 months (*P* = 0.77) for the PKP and DALK groups, respectively, in the matched data. After CEM was performed, the PKP and DALK groups were comparable in terms of the following demographic parameters: recipient and donor age and sex, and other baseline clinical factors like past surgical history; history of VKC, herpes simplex keratitis, and Down syndrome; ocular surface disorders like dry eye disease (DED); history of trauma; and the presence and severity of corneal neovascularization. Moreover, after matching, the quality of donor grafts was comparable between the PKP and DALK groups.

After CEM, the most common suture technique in the DALK group was the single-interrupted method (56.7%), while the combined method was the most frequently used (53.1%) in the PKP group. The difference in the type of suture used in the two groups was not statistically significant (*P* = 0.13). Before CEM, graft opacity was present in 29.9% of patients in the DALK group and 15.1% in the PKP group (*P* = 0.03). After that, however, it changed to a comparable rate of 25.4% and 16.4%, respectively (*P* = 0.35).

#### Visual outcomes 

BCVA significantly improved after keratoplasty in both groups (*P <* 0.001 for all comparisons). Postoperative BCVA was not significantly different in the two groups [Table 4]. While the Logarithm of the Minimum Angle of Resolution (LogMAR) measurements of visual acuity were significantly higher in the PKP group (*P* = 0.01), the postoperative measurements showed statistically similar results (*P* = 0.75), implying the higher efficacy of PKP in recovering visual acuity. However, after CEM, the baseline difference between the two groups was no longer significant (*P = *0.09), and both techniques yielded similar results (*P* = 0.48), which contradicts the earlier finding. Postoperative BCVA ≥ 20/40 was observed in 79.0% and 70.8% of the eyes after DALK and PKP, respectively (*P = 0.28*).

#### Safety and complications 

After CEM, suture-related complications developed in 19 eyes (28.4%) in the PKP group and 16 eyes (23.9%) in the DALK group (*P = 0.87*), a difference that remained insignificant after adjusting for donor quality (*P* = 0.35). There was no statistically significant difference between the study groups in the incidence of any type of suture complication. No graft ulcer or failure developed because of suture-related complications. Table 5 compares the complications between the two groups.

#### Graft rejection and failure 

After CEM, a minimum of one graft rejection episode was observed in 16 eyes of the PKP group and 6 eyes of the DALK group during the study period (*P *= 0.02). The median interval from the corneal transplantation to the first rejection episode was 8.5 months (range, 1–48 months) after PKP and 22.5 months (range, 1–70 months) after DALK (*P *= 0.65). Only seven patients before matching and three patients after matching, all in the PKP group, encountered more than one episode of graft rejection (*P* < 0.001). The cumulative number and the type of rejection episodes are summarized in Table 3. One endothelial, one stromal, and one subepithelial graft rejection in the PKP group resulted in graft failure.

#### Comorbidities and possible etiologies of graft rejection 

Before CEM, the most predominant comorbidities of graft rejection after PKP were graft vascularization (*n* = 10), followed by infection (*n* = 4), graft infiltration, and glaucoma (each *n* = 2), stitch abscess, and dry eye (each *n* = 1). The comorbidities and possible etiologies** of 17 (44.7%) cases of rejection remained unidentified in the PKP group. In the DALK group, interface haziness—which was attributed to intraoperative interface hemorrhage or lipid extravasation due to donor–recipient junctional vascularization—was the most common cause (*n* = 9), followed by dry eye (*n* = 2), infection, and loose suturing (each, *n* = 1). The etiology of 1 case remained unknown. After matching was performed, the comorbidities and possible etiologies for graft rejection after PKP were infection (*n* = 3), graft vascularization (*n* = 2), graft infiltration, glaucoma (each, *n* = 2), stitch abscess, and dry eye (each, *n* = 1). In the DALK group, these were interface haziness (*n* = 3), followed by dry eye, infection, and loose suturing (each, *n* = 1).

Graft failure was observed in six eyes in the PKP group and eight eyes in the DALK group. After CEM, these values changed to three and four in these groups, respectively (*P *= 0.52). The cumulative probability of graft survival was 96.7%, 94.8%, and 94.8% in the PKP group at one-, three-, and five-year follow-ups, respectively. In comparison, the survival rate was 98.5%, 98.5%, and 87.1% in the DALK group for these follow-ups, respectively (*P *= 0.71). The mean duration of graft survival was 35.4 months in the PKP group and 35.2 months in the DALK group [Table 6].

#### Comorbidities and possible etiologies of graft failure 

Before CEM, the most common causes associated with graft failure after PKP were graft vascularization and infection (each, *n* = 2, 33.3%), followed by dry eye and trauma (each, *n* = 1, 16.7%). In the DALK group, interface haziness was the leading cause (*n* = 7, 87.5%), followed by infection (*n* = 1, 12.5%). After CEM, the causes consisted of infection (*n* = 2, 66.7%) and dry eye (*n* = 1, 33.3%) in the PKP group, and interface haziness (*n* = 3, 75%) and infection (*n* = 1, 25%) in the DALK group.

#### Factors associated with corneal graft rejection and failure

On univariate analysis, the strongest risk factor predictive of graft rejection was corneal vascularization in both the PKP group (Hazard ratio [HR] = 5.69, 95% CI, 1.75 to 18.49, *P *= 0.004; log-rank *P* < 0.01) and the DALK group (HR = 6.75, 95% CI, 1.12 to 40.55, *P *= 0.04, log-rank *P* = 0.02), followed by recipient age only in the PKP group (HR = 1.06, 95% CI, 1.02 to 1.11, *P =* < 0.01, log-rank *P* = 0.04) and DED only in the DALK group (HR = 3.45, 95% CI, 1.23 to 9.66, *P *= 0.02, log-rank *P* = 0.02).

**Figure 1:**
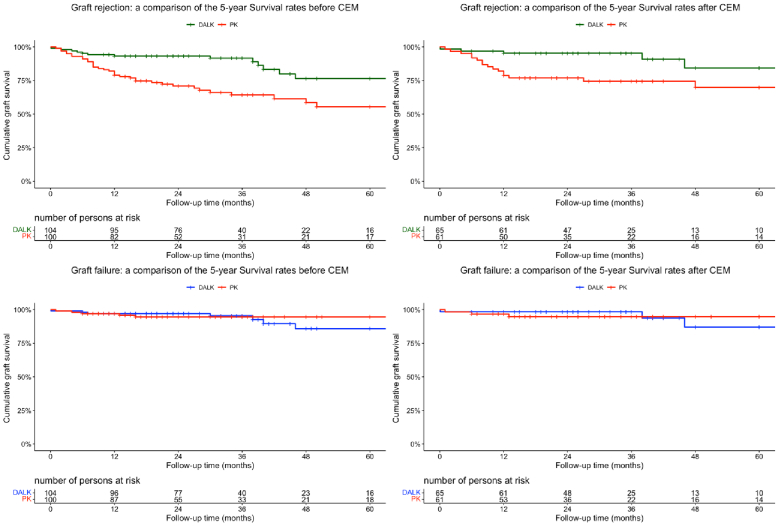
Kaplan Meier graphs depicting the graft survival rates after deep anterior lamellar keratoplasty (DALK) versus penetrating keratoplasty (PKP) before and after 1:1 coarsened exact matching (CEM).

On multivariate Cox regression and after testing multiple combinations of covariates, the model adjusted for only corneal vascularization and recipient age performed the best and PKP was determined to be an independent predictor of graft rejection (HR = 3.19, 95% CI, 1.23 to 8.29, *P *= 0.02), but not graft failure (HR = 0.87, 95% CI, 0.18 to 3.98, *P *= 0.86). Figure 1 illustrates the Kaplan–Meier survival curves comparing graft survival between DALK and PKP before and after matching.

#### Dry eye disease 

Interestingly, the emergence and severity of DED were associated with a higher rate of graft rejection in the DALK group. Experiencing mild dry eye symptoms compared to normal status in the DALK group was associated with a meaningful, yet not statistically significant 4.38-fold increase in the risk for graft rejection (HR: 4.38, 95% CI, 0.26 to 72.62, *P *= 0.30), which could be due to the low statistical power of our study. Additionally, moderate to severe symptoms (compared to normal subjects) were associated with a clinically meaningful and statistically significant increase (nearly 12.5-fold) in the occurrence of graft rejection (HR: 12.60, 95% CI, 1.39 to 114.00, *P *= 0.02). On the other hand, in the PKP group, such an effect was not observed (*P* = 0.97). The proportion of dry eye symptoms and the association of their severity with graft rejection are shown in Table 7.

#### Seasonal association with graft rejection

After CEM, the highest rate of graft rejection occurred in the summer (66.7%) for the DALK group and in summer and autumn (both 25%) for the PKP group. However, there was no significant difference in the seasonal occurrence of graft rejection between DALK and PKP (*P* = 0.30).

##  DISCUSSION

The current study compared the efficacy and safety indices of PKP versus DALK in patients with KCN utilizing a 1:1 CEM method. We primarily aimed to shed further light on the difference in graft survival between the two groups and identify potential risk factors through univariate and multivariate analyses. The results of the present study demonstrated that while DALK and PKP exhibit a comparable BCVA and complication rate, the latter is associated with a lower graft survival rate in a five-year follow-up period.

Several risk factors can contribute to graft rejection. Graft rejection occurs when the recipient's immune system recognizes the transplanted cornea as foreign and mounts an immune response against it. The presence of corneal neovascularization, repeated keratoplasty, a history of previous graft rejection and failure, the type of diagnosis requiring corneal transplantation, and potentially an older donor age ( ≥ 80) are among the previously identified causes of graft rejection and failure in PKP.^[28]^ However, in the present study, KCN was the only diagnosis leading to keratoplasty, and all patients underwent their first keratoplasty.

Since corneal vascularization was not present at the time of patient recruitment, we matched patients who later developed corneal neovascularization. Various intrinsic factors might affect the formation of corneal neovascularization. As neovascularization is an independent risk factor for graft failure and rejection, not necessarily caused directly by the surgical method, we used a technique to adjust the two groups of PKP and DALK for neovascularization.^[20,29]^

The results of the present study suggest an association between DED and graft rejection in the DALK group. On the other hand, this effect was not observed in the PKP group. This finding can be attributed to two factors: (1) the higher rate of suture-related complications in patients with DALK compared to PKP (although not present in this study)^[30]^ and (2) the inflammatory component of DED that can bring immune cells to the corneal interface.^[31]^ The same results were not observed in patients undergoing PKP. This finding could be attributed to the study's lower statistical power in identifying this pattern in the PKP group, the better ocular surface status in these patients, and possibly the fact that the host immune response has already been activated after PKP, regardless of the impact of DED.

On multivariate analysis, PKP emerged as a separate risk factor for graft rejection, but not graft failure. This finding was in line with the results of a systematic review and meta-analysis by Liu et al, which showed a significantly higher rate of graft rejection in the PKP group compared to the DALK group (OR = 0.28, 95% CI, 0.15 to 0.50, *P* < 0.001). In terms of graft failure, the results were comparable between the two groups (OR = 1.05, 95% CI, 0.81 to 1.36, *P* = 0.73).^[32]^

The rate of five-year graft rejection after PKP (30.1%) and DALK (15.7%) observed in our study is in line with the previously reported range (5.8% to 41%).^[30]^ Graft survival rate is one of the most important indicators to assess the efficacy of the corneal transplantation procedure. Graft survival has been reported to be favorable in KCN, with averages of 97% and 90% at five-year intervals after PKP and DALK, respectively.^[30]^ We observed comparatively lower rates of graft survival in both groups (five-year graft survival rates of 94.8% for PKP and 87.1% for DALK). This could be due to our definition of graft failure and the relatively high proportion of surgeries performed by corneal fellows in the early stages of their training (41.8% in the PKP group and 37.3% in the DALK group). However, after CEM, surgeon type was not associated with a meaningful or significant difference in graft survival rates (HR for graft rejection: 1.03, 95% CI, 0.44 to 2.41, *P* = 0.95; HR for graft failure: 1.14, 95% CI, 0.26 to 5.11, *P* = 0.86).

Immune graft rejection occurred more frequently in PKP, but it did not affect the mid-term survival. This vast difference in reported outcomes may be due to differences in the definition of rejection. Other reasons for the difference in rejection rates may include the duration of postoperative follow-up or the initial cause of transplantation.

Regarding efficacy and visual outcomes, our study revealed that both methods provide a significant improvement, yet statistically similar postoperative BCVA. Considering the significant baseline difference in BCVA between the two groups, it may initially seem that PKP could provide better results. Interestingly, after CEM, the remarkable baseline difference in the patients' BCVA between the two groups disappeared, and the postoperative BCVA was still comparable between the two groups. This observation confirms that both methods yielded similar efficacy, which is in agreement with previous studies. Consistent with the present study, all three previous clinical trials comparing big-bubble DALK and PKP in KCN have concluded that both techniques demonstrate similar visual outcomes.^[18,33,34]^ BCVA results in DALK might be less favorable when the surgeon is in the early stages of experience, but more optimal results could be achieved with more experience to bare the Descemet membrane or keep the thinnest posterior stromal residue. More recent studies,^[18,35]^ unlike earlier ones,^[36,37]^ are compatible with our results in terms of the observed visual improvement.

In summary, the DALK and PKP groups were comparable regarding the postoperative BCVA, suture complications, incidence of postoperative uveitis, postoperative glaucoma, and ocular surface changes (e.g., postoperative DED). However, the presence and higher severity of DED were significantly associated with higher rates of graft rejection only in the DALK group. The risk of graft rejection was higher among patients undergoing PKP, while the two groups showed similar graft failure rates. It should be added that corneal vascularization was identified as the sole potential contributor to graft failure.

##  Financial Support and Sponsorship

None.

##  Conflicts of Interest

None.
